# Feasibility and Psychometric Properties of the Infant Toddler Quality of Life (ITQOL) Questionnaire in a Community-Based Sample of Healthy Infants in China

**DOI:** 10.1007/s10995-018-2439-8

**Published:** 2018-02-03

**Authors:** Sheri Volger, Jeanne M. Landgraf, Meng Mao, John Ge, Robert Northington, Nicholas P. Hays

**Affiliations:** 1 Clinical Research, Research and Development, Nestlé Nutrition, 3000 Horizon Drive, King of Prussia, PA 19406 USA; 2 HealthActCHQ, Inc. (HACHQ), 800 Boylston Street, 16th Floor, Boston, MA 02199 USA; 3 Chengdu Women’s and Children’s Central Hospital, Chengdu, China No. 1617, Riyue Avenue, Chengdu, 610091 Sichuan Province People’s Republic of China; 4 Wyeth Nutritional (China) Company Ltd., 30F, CITIC Square, 1168 Nanjing West Road, Shanghai, China

**Keywords:** Infant toddler quality of life (ITQOL), Maternal quality of life, Health-related quality of life, China, Questionnaires

## Abstract

*Objective* Evaluate the feasibility and psychometric properties of the Infant Toddler Quality of Life (ITQOL) questionnaire as a measure of health-related quality of life (HRQOL) in a sample of Chinese infants. *Methods* The linguistically validated Simplified Chinese version of the ITQOL was used in a multicenter, observational study of healthy, term infants (N = 427), age 6 weeks at enrollment, in China. At Days 1 and 48, parents/guardians completed the ITQOL, the Short Form Health Survey (SF-12v2) and the Infant Gastrointestinal Symptom Questionnaire (IGSQ). ITQOL feasibility, reliability, ceiling/floor effects, concurrent validity and discriminatory validity were evaluated. *Results* Feasibility of administering the ITQOL was supported by strong response rates (> 97%) with < 1% missing items for all scales except physical abilities. Reliability was acceptable (Cronbach’s alpha > 0.70) for all scales except Day 1 General Health (0.67). Floor effects were minimal (< 2%), except Day 1 physical abilities (7%). Ceiling effects increased from Days 1 to 48 across all scales. Concurrent validity was demonstrated by correlations between ITQOL infant-focused scales and IGSQ score (r = −0.20 to − 0.34, p < 0.001) and between parent-focused scales and SF-12v2 mental health composite (MCS) scores (r = 0.29–0.46, p < 0.001). ITQOL scales discriminated between infant subgroups based on illness-related outcomes (sick visits, adverse events) and between parent subgroups based on SF-12v2 MCS scores. *Conclusion* The Simplified Chinese version of the ITQOL performed well in a community-based sample of Chinese infants, with evidence supporting the instrument’s feasibility, reliability, and validity. These data support the ITQOL as a valuable tool to assess HRQOL in Chinese infants.

## Significance

The measurement of health-related quality of life (HRQOL) in infants presents many challenges. The Infant Toddler Quality of Life questionnaire (ITQOL) is a parent-reported measure of HRQOL that was developed using a conceptual framework based on the World Health Organization’s definition of health and developmental guidelines used by pediatricians. The present study is the first to evaluate and present findings that support the feasibility and psychometric properties of the Simplified Chinese version of the ITQOL in a sample of young infants in China.

## Introduction

Infant health-related quality of life (HRQOL) is a multifactorial construct of infant well-being that can provide useful information about an infant’s overall health and development. Consistent with the World Health Organization’s definition of health (i.e., a state of complete physical, mental and social well-being and not just absence of disease) (World Health Organization 1948), the measurement of infant HRQOL in clinical and community research settings can provide valuable information about the physical, mental and social well-being of infants (Ravens-Sieberer et al. 2006), which can then be used to develop a more comprehensive understanding of changes in health status over time or differences between groups. Although reliable and valid measures of HRQOL have been used since the late 1990s in clinical research in school-age children, there are relatively few robust HRQOL instruments specifically designed for and validated in infants, despite mounting interest in quality of life (QOL) information in this population. Moreover, most of the available pediatric HRQOL instruments were developed for use in Western countries and are based on the English language, potentially limiting their utility in cultures with pictorial-based written languages such as in China and Japan.

The Infant Toddler Quality of Life questionnaire (ITQOL) (Landgraf 1994; Raat et al. 2007; Spuijbroek et al. 2011) is a generic parent-reported measure of HRQOL that was developed using a conceptual framework based on the World Health Organization’s definition of health (World Health Organization 1948) and developmental guidelines used by pediatricians (Caplan and Caplan 1993; Caplan 1988; American Academy of Pediatrics 1998). The instrument is intended for use with infants and toddlers no younger than 2 months of age to allow time for parents to adjust to and “get to know” their infants. ITQOL items assess parents’ perceptions of their infants’ overall health, physical functioning, growth and development, bodily pain, temperament and moods, general behavior and general health. As parental worry and concern may affect reporting on child well-being (Darcy et al. 2011), the ITQOL also assesses the degree of worry or anxiety that the parent feels concerning the child’s physical, emotional, cognitive and social development, the degree to which these concerns impact the parent’s time to attend to personal needs, and the parent’s rating of how well the family is getting along with one another.

The ITQOL has been translated into more than 30 languages using rigorous international guidelines (Wild et al. 2005) to ensure the quality of these cross-cultural adaptions. Recently, the ITQOL was used in a multicenter, prospective, observational study (NCT01370967) of infant feeding practices, stooling, gastrointestinal (GI) symptoms and HRQOL in a community-based sample of healthy, young Chinese infants approximately 6 weeks of age at enrollment. Overall, the study found high levels of HRQOL in breastfed, formula-fed and mixed-fed (i.e., both breastfed and formula-fed) infants, with only a few small differences in HRQOL observed between groups (Hays et al. 2016). As this observational study was the first to use the Simplified Chinese version of the ITQOL, and because parents completed the ITQOL twice during the study, the data provided an opportunity to evaluate the feasibility of administering and completing the ITQOL (time needed to complete, response rate, missing item analysis) and the psychometric properties (reliability [Cronbach’s alpha], floor and ceiling effects, concurrent and discriminative validity) of the ITQOL in a sample of healthy Chinese infants. The development of parent-reported outcome (PRO) measures, such as the ITQOL, is an iterative process, with each administration adding to a growing body of empirical evidence related to the instrument’s feasibility, reliability and validity. Appraisal of these measurement properties is essential to assuring that a questionnaire is sufficiently robust for use across an array of settings, cultures, and health conditions.

## Methods

The main objective of the analysis reported here was to evaluate the feasibility and psychometric properties of the Simplified Chinese version of the ITQOL using data from a study conducted at 24 sites in China from September 2011 to June 2013 (Hays et al. 2016; Mao et al. 2018). The majority of study centers were located on China’s East Coast (84%) with additional sites in Central (4%) and Western (12%) regions; over two-thirds of the sites were within public hospitals, eight of which had academic affiliations. Detailed descriptions of the study design, inclusion criteria and main HRQOL results have been published elsewhere (Hays et al. 2016). Briefly, infants were recruited and enrolled into the study at the time of their 6-week well-baby clinic visit. Eligibility criteria included being approximately 42 days old, healthy, term, singleton birth, WHO weight-for-age percentile ≥ 5 and ≤ 95, and parent/guardian (hereafter “parent”) able to comply with the study visits and procedures. Infants were maintained on their parent-selected, pre-study feeding regimens throughout the course of the observational study. Parents completed the ITQOL during study visits on Day 1 (enrollment) and 48. Questionnaires to assess parental HRQOL and infant feeding tolerance were also completed at these study visits, in the same sequence at each visit. A questionnaire to obtain demographic information was completed by parents on Day 1. A study-wide initiation meeting and individual site initiation meetings were held to instruct staff on study procedures including the correct administration of questionnaires. The study was approved by the ethics committee at each site and all procedures met the ethical standards laid down in the 1964 Declaration of Helsinki and its later amendments. Informed consent was obtained from all parents prior to inclusion of the infants in the study.

### Instruments

Data from Simplified Chinese versions of three questionnaires were used in this analysis: the ITQOL, which assessed infant HRQOL, the 12-item Short-Form Health Survey (SF-12v2) (Ware et al. 1996) which assessed parent HRQOL, and the Infant GI Symptom Questionnaire (IGSQ) (Riley et al. 2015) which assessed infant’s feeding tolerance and GI symptom burden.

The ITQOL is a 97-item, self-administered, parent-reported infant HRQOL instrument for children ages 2 months to 5 years. The measure is comprised of 10 infant-focused and 3 parent-focused concept scales, scored on a Likert-type response continuum (e.g., very satisfied to very dissatisfied) (Table 1). Each concept scale begins with a stem question (e.g., How satisfied are you with your child’s…?) followed by item sub-phrases (e.g., physical growth and development, feeding/nursing/eating habits, sleep habits) evaluating the scale’s concept (e.g., Growth and Development, Temperament and Moods). The ITQOL was purposefully designed with stem questions and item sub-phrases (as opposed to separate questions) to reduce respondent burden. In accordance with guidelines for completing the questionnaire, 29 items are purposely skipped in children < 1 year of age (Table 1). The recall period is 4 weeks. For multi-item scales, completion of ≥ 50% of the items within the scale is required to calculate a score ranging from 0 (worst) to 100 (best) (HealthActCHQ 2008).

**Table 1:** Infant Toddler Quality of Life Questionnaire

ITQOL concepts	Number of items
Infant/child-focused	
Overall health (OH)	1
Physical abilities (PA)	10
Growth and development (GD)	10
Bodily pain/discomfort (BP)	3
Temperament and moods (TM)	18
Behavior overall	12^a^
Global behavior	1^a^
Getting along	15^a^
General health (GH)	11
Change in health	1^a^
Parent/family-focused	
Emotion impact (PE)	7
Time impact (PT)	7
Family cohesion (FC)	1

*ITQOL* Infant Toddler Quality of Life Questionnaire

^a^Items are skipped for infants < 1 year of age

HRQOL of the parents was measured with the SF-12v2, a shortened, 12-item version of the 36-item Short-Form Health Survey (Ware et al. 1996). It is a self-administered instrument, from which 2 composite scores, a physical health composite summary (PCS) and a mental health composite summary (MCS), are derived. Higher PCS and MCS scores indicate better functional physical and mental health, respectively (Saris-Baglama et al. 2010). The standard 4-week recall form was administered.

The IGSQ is a 13-item validated interviewer-administered parent questionnaire which assesses from the parent’s perspective how well an infant tolerates his or her feeding regimen and the infant’s overall GI symptom burden (Riley et al. 2015). A summary GI burden index score is calculated. The recall period is the previous 7 days and the information is collected with a 5-point Likert-scale assessing five GI symptom domains: stooling, spitting up/vomiting, flatulence, crying, and fussiness. IGSQ summary scores range from 13 to 65, with lower scores corresponding with better parent-perceived feeding tolerance and less GI distress.

### ITQOL Translation and Administration

The ITQOL was linguistically translated into Simplified Chinese using rigorous international guidelines (Wild et al. 2005; U.S. Department of Health and Human Services, Food and Drug Administration 2015). Forward and back translations were reconciled and harmonized and cognitive debriefing was conducted with in-country Chinese parents prior to finalizing the translation.

During the study design process, the research team engaged local professionals and collaborated with the sites to adapt research methodologies to local clinical and cultural practices. Adaptations included modification of the study visit schedule to coincide with well-baby clinic visits, inclusion of a sufficiently wide visit window to avoid scheduling study visits during national holidays and school vacations, the presence of a study nurse during assessment visits to hold and care for infants while parents completed questionnaires, and provision of blankets to each site to ensure infant warmth during study visits. For participating in the study, each parent received a book about infant care (value approximately $5 USD) and, at some sites, parents attended a hospital-sponsored baby care class.

### Statistical Analyses

Data were analyzed to evaluate the feasibility, reliability and concurrent and discriminative validity of the ITQOL using SAS®9.1.3.Software. Normality was tested and non-parametric tests were applied as appropriate. ITQOL feasibility was evaluated using data on: ease of administration (mean completion time; number and evaluation of questions asked by parents); response rate (percent questionnaires completed), and missing item rates (number of items left blank/unanswered) at Days 1 and 48. Median score and inter-quartile range were calculated for each scale. ITQOL reliability was evaluated using Cronbach’s alpha for all scales with more than one item. The a priori threshold for adequate reliability was alpha ≥ 0.70 (Cronbach 1951). Floor and ceiling effects (percentage of respondents with scores in the lower and upper quartiles) were also assessed.

Concurrent validity of the ITQOL was assessed by comparing the infant-focused scale scores with IGSQ scores, and the ITQOL parent-focused scale scores with the SF-12v2 MCS. It was hypothesized that infants tolerating their feedings and experiencing fewer GI symptoms would be perceived by their parents as having better infant HRQOL. It was also hypothesized that better parental mental functioning (SF-12v2 MCS) would be associated with better (higher) parent-focused scale scores on the ITQOL. Correlations were interpreted as < 0.3 = small; 0.3–0.5 = moderate; >0.5 = large (Cohen 1988).

Discriminative validity of the ITQOL was assessed using the Wilcoxon rank sum test to compare infant-focused scale scores in subgroups of infants based on number of visits to a health care provider (HCP) (0; 1; ≥ 1; > 2) and number of parent-reported adverse events (AEs) (0; ≥ 1; and ≥ 2). Discriminative validity also was evaluated by comparing ITQOL parent-focused scale scores in subgroups of parents based on mental health scores derived from the SF-12v2 MCS scores (worse mental health ≤ 45; average mental health > 45 to  <60; better mental health ≥ 60). Effect size (ES) was calculated (ES = mean_1_ − mean_2_/SD mean_2_) to compare subgroup scores. An ES of ≥ 0.5 was used as the threshold for a minimally important difference (Norman et al. 2003).

## Results

### Participants

A total of 427 (97%) of the 440 enrolled participants completed all study questionnaires at both Day 1 and 48 and were included in this analysis. Participant characteristics are shown in Table 2. In general, the infants had a mean (SD) age of 42.3 (3.5) days at enrollment, with nearly 55% born via cesarean section and 86% were the first-born child. None were being cared for outside the home. Mean (SD) maternal age was 29.5 (3.8) years. Parents were mostly well-educated, with high rates of full-time employment.

**Table 2:** Participant characteristics at time of study enrollment

Participant characteristics	Total N = 427
Infants	
Age (days)	42.3 ± 3.5
Gestational age (weeks)	38.9 ± 1.0
Sex (male)	241 (56.4)
Race (Asian)	427 (100.0)
Birth order (first-born)	367 (86.0)
Infant attends day care	0 (0.00)
Mothers	
Age (years)	29.5 ± 3.8
C-section delivery	233 (54.6)
Marital status (married)	427 (100.0)
Education (years)	15.2 ± 2.9
Education (highest level)	
High school diploma	58 (13.6)
College degree	60 (14.1)
Graduate degree	279 (65.3)
Occupation	
Employed full-time	348 (81.5)
Professional (e.g. teacher, physician)	148 (34.7)
Service/shop/market sales	66 (15.5)
Homemaker	76 (17.8)
Fathers	
Education (years)	15.2 ± 2.9
Employed full-time	410 (96.0)
Household	
Number of adults living in the household	3.1 ± 1.1

Data presented are mean ± standard deviation or number (%)

### ITQOL Scores

Table 3 shows median ITQOL scale scores and interquartile ranges at Days 1 and 48. Median scores for all infant and parent-focused scales except temperament and moods (TM) were ≥ 83. At Day 48, all median scale scores were either stable or had increased up to 7 points.

**Table 3:** Total ITQOL scores, floor and ceiling effect, and Cronbach’s alpha at Day 1 and 48

ITQOL concepts	N	Median	Inter-quartile range	Floor^a^ (%)	Ceiling^b^ (%)	Cronbach’s alpha
Infant-focused concepts						
Overall health (OH)						
Day 1	426	85	60–85	0.05	64.1	na^c^
Day 48	416	85	60–85	0.0	67.6	na^c^
Physical abilities (PA)						
Day 1	42	93	73–100	7.1	71.4	na^c^
Day 48	146	100	83–100	2.1	84.9	0.94
Growth and development (GD)						
Day 1	424	83	70–95	0.0	67.7	0.88
Day 48	416	88	75–98	0.2	76.7	0.89
Bodily pain/discomfort (BP)						
Day 1	426	100	92–100	0.0	92.3	0.81
Day 48	416	100	92–100	0.2	96.2	0.79
Temperament and moods (TM)						
Day 1	424	72	67–78	0.0	40.1	0.74
Day 48	415	76	71–83	0.0	60.7	0.76
General health (GH)						
Day 1	426	84	75–89	0.0	78.2	0.67
Day 48	413	84	77–91	0.0	82.8	0.73
Parent/family-focused concepts						
Emotion impact (PE)						
Day 1	426	93	79–96	1.4	85.0	0.92
Day 48	413	93	86–100	1.2	91.3	0.91
Time impact (PT)						
Day 1	427	90	81–95	1.4	81.3	0.92
Day 48	412	95	86–100	0.7	86.4	0.88
Family cohesion (FC)						
Day 1	427	85	60–85	0.5	68.9	na^c^
Day 48	412	85	60–93	0.0	69.4	na^c^

*ITQOL* Infant Toddler Quality of Life Questionnaire, *na* not applicable

^a^% of respondents with scores in the lowest quartile

^b^% of respondents with scores in the highest quartile

^c^Cronbach’s alpha cannot be calculated for OH and FC, which are assessed with single items; could not calculate Cronbach’s alpha for PA at Day 1 due to low response rate for the items in this scale on Day 1 (infants not yet rolling over, sitting up, etc) and a lack of variance in the non-missing responses

### Feasibility

#### Ease of Administration

The ITQOL took less than 10 min to complete and participants asked very few questions during completion. The majority of questions were related to skip patterns for the omitted items that were not applicable to this study population.

#### Response Rate

The ITQOL was completed by 100% of participants at Day 1 and 97% of participants at Day 48.

#### Missing Item Analysis

The percentage of missing items at both visits was < 1% for all ITQOL scales except for Day 1 physical abilities (PA) with > 77% of items reported as “not doing yet” (e.g., sitting up, crawling, taking steps/walking). Thus, PA scores were based on only 42 and 146 infants at Day 1 and 48, respectively.

### Floor and Ceiling Effects

ITQOL data were non-normally (left-skewed) distributed (Shapiro-Wilks test, p < 0.001 for all scales) and analyzed using non-parametric tests. Floor effects (Table 3) were minimal and remained stable over time and across scales with the exception of PA. In contrast, there was an increase in the ceiling effect across all scales at Day 48.

### Reliability

Cronbach’s alpha was > 0.70 at Days 1 and 48 for all multi-item scales except General Health (GH) (0.67), which nearly reached the a priori threshold (0.70) for reliability. In addition, Cronbach’s alpha could not be calculated for PA at Day 1 due to the low response rate (n = 42) for these items and low variability in the non-missing responses (between 66 and 96% reported no limitation).

### Concurrent Validity

Correlations evaluating concurrent validity are reported in Table 4. At Day 1, the growth and development (GD) and bodily pain (BP) scales were significantly and moderately negatively correlated with IGSQ scores, while three additional infant-focused scales [overall health (OH), TM, and GH] were significantly but weakly correlated with IGSQ scores. At Day 48, the correlation between IGSQ and OH scores was no longer significant and all correlations were weakly negative.

**Table 4:** Correlations between ITQOL infant-focused concept scores and IGSQ score and between ITQOL parent/family-focused concept scores and SF-12v2 MCS score at Day 1 and 48

ITQOL concepts	Spearman’s rho (P value)
Infant-focused concepts	Correlation with IGSQ Score
Overall health (OH)	
Day 1	− 0.21 (< 0.001)
Day 48	− 0.07 (0.182)
Physical abilities (PA)	
Day 1	− 0.06 (0.714)
Day 48	0.09 (0.301)
Growth and development (GD)	
Day 1	− 0.30 (< 0.001)
Day 48	− 0.19 (< 0.001)
Bodily pain/discomfort (BP)	
Day 1	− 0.34 (< 0.001)
Day 48	− 0.17 (< 0.001)
Temperament and moods (TM)	
Day 1	− 0.26 (< 0.001)
Day 48	− 0.20 (< 0.001)
General health (GH)	
Day 1	− 0.20 (< 0.001)
Day 48	− 0.15 (0.002)
Parent/family-focused concepts	Correlation with SF-12v2 MCS Score
Emotion impact (PE)	
Day 1	0.42 (< 0.001)
Day 48	0.49 (< 0.001)
Time impact (PT)	
Day 1	0.46 (< 0.001)
Day 48	0.40 (< 0.001)
Family cohesion (FC)	
Day 1	0.29 (< 0.001)
Day 48	0.37 (< 0.001)

*ITQOL* Infant Toddler Quality of Life Questionnaire, *IGSQ* Infant GI Symptom Questionnaire, *SF-12v2 MCS* Short-Form Health Survey-12v2 Mental Health Composite Summary

At Day 1, significant correlations were observed between parent SF-12v2 MCS scores and all three parent-focused ITQOL scales. Positive, moderate correlations were found with parent emotion-impact (PE) and parent time-impact (PT) scales, whereas a weaker positive correlation was found with family cohesion (FC). By Day 48, positive, moderate correlations were observed between SF-12v2 MCS scores and all parent-focused ITQOL scales.

### Discriminative Validity

Discriminative validity was evaluated by comparing ITQOL scale scores among subgroups of infants classified according to number of sick visits made to health care providers (HCPs) (Table 5) and incidence of adverse events (Table 6). In general, infants with one or more HCP visit had significantly lower ITQOL scale scores, with the exception of PA, GD, and FC scores. The effect size was ≥ 0.5 for four of the six infant-focused scales. A similar pattern was seen with adverse events. Discriminative validity of the parent-focused scale scores also was evaluated by comparing scale scores among subgroups of parents based on SF-12v2 MCS scores (Table 7). Mean scores for ITQOL parent-focused scales were significantly worse (lower) in the subgroup of mothers with the lowest mental health scores (i.e., lower SF-12v2 MCS scores) compared to the subgroup with the best mental health scores (i.e., higher SF-12v2 MCS scores).

**Table 5:** Discriminative validity of the ITQOL scale scores among subgroups of infants defined by number of sick visits to health care providers at Day 48

	Number of visits to health care provider per subject				Effect size^a^ and P value for pairwise comparison^b^					
	0	1	≥ 1	> 2	0 vs. 1		0 vs. ≥ 1		0 vs. > 2	
					Effect size^a^	P value	Effect size^a^	P value	Effect size^a^	P value
Infant-focused concepts										
Overall health (OH)										
N	364	43	52	3	0.4	0.012	0.5	< 0.001	0.8	0.086
Mean (SD)	80.1 (15.5)	72.1 (20.1)	68.8 (21.0)	58.3 (27.5)						
Physical abilities (PA)										
N	130	14	16	1	0.1	0.638	0	0.757	–	–
Mean (SD)	88.0 (19.0)	86.6 (25.8)	87.8 (24.3)	93.0 (0)						
Growth and development (GD)										
N	364	43	52	3	0.2	0.162	0.3	0.051	0.6	0.276
Mean (SD)	84.9 (14.4)	81.6 (15.2)	80.4 (16.1)	78.7 (11.0)						
Bodily pain/discomfort (BP)										
N	364	43	52	3	0.5	< 0.001	0.6	< 0.001	1.2	0.002
Mean (SD)	95.5 (8.7)	87.6 (15.8)	85.4 (16.0)	75.0 (17.0)						
Temperament and moods (TM)										
N	363	43	52	3	0.3	0.027	0.3	0.024	− 0.2	0.576
Mean (SD)	76.4 (8.9)	73.9 (7.5)	74.0 (7.5)	78.3 (11.5)						
General health (GH)										
N	361	43	52	3	0.5	0.002	0.6	< 0.001	0.7	0.145
Mean (SD)	84.2 (11.0)	78.8 (10.6)	77.1 (11.7)	72.3 (17.0)						
Parent/family concepts										
Emotion impact (PE)										
N	361	43	52	3	0.5	< 0.001	0.5	< 0.001	0.3	0.268
Mean (SD)	90.3 (14.6)	83.2 (14.9)	82.8 (15.2)	88.0 (7.2)						
Time impact (PT)										
N	360	43	52	3	0.3	0.007	0.4	< 0.001	0.4	0.280
Mean (SD)	89.6 (14.5)	83.8 (16.9)	82.7 (17.8)	84.0 (14.9)						
Family cohesion (FC)										
N	360	43	52	3	0.2	0.173	0.2	0.39	− 1.1	0.367
Mean (SD)	80.2 (17.7)	75.8 (19.6)	77.0 (20.3)	90.0 (8.7)						

*ITQOL* Infant Toddler Quality of Life Questionnaire, *SD* standard deviation

^a^Effect size = mean (0 visits) − mean (1, ≥ 1, or > 2 visits)]/SD (for 1, ≥ 1, or > 2 visits)

^b^Wilcoxon test was used for comparison

**Table 6:** Discriminative validity of the ITQOL scale scores among subgroups of infants defined by the number of episodes of illnesses (based on adverse events) at Day 48

	Number of adverse events per subject			Effect size^a^ and P value for pairwise comparison^b^			
	0	≥1	≥2	0 vs. ≥ 1		0 vs. ≥ 2	
				Effect size^a^	P value	Effect size^a^	P value
Infant-focused concepts							
Overall health (OH)							
N	344	72	12	0.5	< 0.001	1.7	< 0.001
Mean (SD)	80.4 (15.3)	70.6 (20.6)	49.6 (18.6)				
Physical abilities (PA)							
N	126	20	4	0.2	0.392	− 0.4	1
Mean (SD)	88.5 (18.6)	84.6 (24.7)	92.3 (9.7)				
Growth and development (GD)							
N	344	72	12	0.3	0.006	0.6	0.038
Mean (SD)	85.2 (14.4)	80.2 (15.3)	74.0 (19.9)				
Bodily pain/discomfort (BP)							
N	344	72	12	0.5	< 0.001	1.1	< 0.001
Mean (SD)	95.6 (8.7)	87.7 (14.7)	78.5 (15.7)				
Temperament and moods (TM)							
N	344	71	12	0.1	0.392	0.6	0.048
Mean (SD)	76.2 (8.9)	75.6 (7.9)	72.2 (6.6)				
General health (GH)							
N	342	71	12	0.4	0.002	0.5	0.062
Mean (SD)	84.2 (11.0)	79.1 (12.2)	75.3 (16.2)				
Parent/family-focused concepts Emotion impact (PE)							
N	342	71	12	0.3	0.004	0.8	< 0.001
Mean (SD)	90.0 (14.9)	86.2 (14.4)	79.4 (13.5)				
Time impact (PT)							
N	341	71	12	0.3	0.005	0.6	0.015
Mean (SD)	89.7 (13.9)	83.9 (19.1)	81.3 (14.2)				
Family cohesion (FC)							
N	341	71	12	0.1	0.462	0.4	0.244
Mean (SD)	80.3 (17.3)	77.2 (21.2)	71.3 (25.1)				

ITQOL Infant Toddler Quality of Life Questionnaire, SD standard deviation

^a^Effect size = mean (0 adverse events) − mean (≥ 1 or ≥ 2 adverse events)]/SD (≥ 1 or ≥ 2 adverse events)

^b^Wilcoxon test was used for comparison

**Table 7:** Discriminative validity by comparing parent-focused ITQOL scale scores among subgroups of parents with low (< 45), average (≥ 45 and < 60), and high (≥ 60) SF-12v2 MCS scores at Day 1 and 48

Parent/family-focused concepts	SF-12v2 MCS scores			Effect size^a^ and P value for pairwise comparison^b^					
	Low	Average	High	Low vs. Average		Low vs. High		Low vs. Average or High	
				Effect size^a^	P value	Effect size^a^	P value	Effect size^a^	P value
Day 1									
Emotion impact (PE)									
N	46	310	69	− 0.6	< 0.001	− 2.8	< 0.001	− 0.8	< 0.001
Mean (SD)	75.4 (22.2)	86.1 (16.5)	95.7 (7.3)						
Time impact (PT)									
N	46	311	69	− 0.8	< 0.001	− 2.3	< 0.001	− 0.9	< 0.001
Mean (SD)	71.1 (23.1)	85.0 (17.4)	94.9 (10.2)						
Family cohesion (FC)									
N	46	311	69	− 0.6	0.056	− 1.2	0.003	− 0.7	0.023
Mean (SD)	68.9 (27.9)	78.9 (17.5)	85.2 (13.4)						
Day 48									
Emotion impact (PE)									
N	28	308	76	− 1.1	0.012	− 3.1	< 0.0001	− 1.2	0.002
Mean (SD)	74.9 (29.7)	88.9 (13.2)	96.5 (6.9)						
Time impact (PT)									
N	28	308	75	− 0.7	0.008	− 2	< 0.0001	− 0.9	0.002
Mean (SD)	77.2 (21.2)	88.2 (14.9)	95.2 (9.2)						
Family cohesion (FC)									
N	28	308	75	− 0.6	0.009	− 1.5	< 0.0001	− 0.7	0.002
Mean (SD)	67.9 (22.6)	79.1 (18.0)	87.1 (12.7)						

*ITQOL* Infant Toddler Quality of Life Questionnaire, *SD* standard deviation, SF-12v2 MCS Short-Form Health Survey-12v2 Mental Health Composite Summary

^a^Effect size = mean (low MCS score) − mean (average, high, or average + high)]/SD (average, high, or average + high)

^b^Wilcoxon test was used for comparison

## Discussion

The measurement of HRQOL in infants presents many challenges. The present study is the first to report on the feasibility, reliability, floor and ceiling effects and validity of a Simplified Chinese translation of the ITQOL in a sample of infants aged 42–90 days from urban and suburban areas across several regions in China. The findings from this study suggest that the ITQOL is reliable and valid for use in studies that enroll healthy, Chinese infants similar to those in the current study.

In addition to meeting rigorous translation standards including cognitive debriefing, it is essential that the applicability of any measure be carefully evaluated in a real-world setting. The majority of studies to date that have used the ITQOL have focused on older infants (≥ 12 months of age). This study demonstrated that it is acceptable, in concert with consideration of local customs and clinical practice, to administer the ITQOL in young infants in China. At enrollment and even at follow up, our study population was much younger than the target age of previous studies (Landgraf 1994; Raat et al. 2007; Spuijbroek et al. 2011; Darcy et al. 2011; Alonso et al. 2008; Bannink et al. 2010; Flink et al. 2013). Even so, less than 1% of ITQOL items of key interest to this study were unanswered. As anticipated, at Day 1, when the infants were approximately 6 weeks old, parents reported the majority of infants as “not doing yet” sitting up, crawling, or taking steps/walking. Although this resulted in limited PA data at Day 1, the ITQOL captured developmentally appropriate improvements for these items at Day 48. Thus, the sensitivity of the scale to detect such changes suggests that it is indeed measuring perceived change in physical abilities.

The reliability of the Simplified Chinese version of the ITQOL was supported by acceptable Cronbach’s alpha coefficients for all scales at Days 1 and 48. High ceiling effects were observed, which was not unexpected given the inclusion of only healthy infants in this study. The ceiling effect was high at Day 1 for all scales and trended further upward at Day 48. The most notable finding was an increase in the ceiling effect for the TM scale, congruent with the infants’ developmental improvements in sleep, feeding, and alertness. Not surprising, the one concept with the greatest floor effect at Day 1 was the PA scale (7.1%). However, the effect decreased to approximately 2% by Day 48, consistent with age-appropriate improvements in physical abilities.

We observed a notable 5-point increase in the median PT score between Days 1 and 48, suggesting that by the time the infants were 3 months of age, parents were adjusting and feeling less limited in the amount of time they had for themselves. This finding is even more striking given the high rate (55%) of cesarean births in this study population [and reported in China by others (Festin et al. 2009; Mi and Liu 2014; Feng et al. 2012; Lumbiganon et al. 1902)], which typically require a longer postnatal recovery period for mothers. Furthermore, the amount of parental anxiety or worry about infant development remained unchanged, as did the family’s ability to get along with one another despite the potential for disruptions and changes in routine that naturally occur after an infant’s birth. The stability of these concepts in our study population might be explained by maternal age, marital status, and/or the presence of extended family support in many homes.

Correlations between parent SF-12v2 MCS scores and ITQOL parent-focused scales supports the validity of the ITQOL. The PE and PT scales were significantly moderately correlated with MCS scores but a weaker, significant correlation was observed with FC. These results are supported by previous studies which have shown a positive association between maternal postpartum depression and negative emotions (Darcy et al. 2011; O’Hara and McCabe 2013), and suggest a relationship between a new parent’s emotional well-being and the degree to which they feel anxious or worried about their infant and how limited they are in attending to their own personal needs. The present findings suggest that the relationships between parent mental health and the emotional and time impacts of parenting are stronger than the relationship between parent mental health and how well the family gets along. This relationship may be due in part to the large number of married, one-child, and extended family households in this present study, which is a cultural factor that warrants further exploration.

Overall, this study demonstrated that it is both feasible and informative to assess infant HRQOL based on parent perception, and this information can then be examined in relationship to other health outcomes. For example, at Day 1, a moderate, negative correlation was observed between IGSQ scores and those ITQOL infant-focused scales that encompass GI functioning: GD (questions about feeding, bowel function) and BP (asks about the amount and frequency of discomfort due to gas, teething, injury or illness and the degree to which pain interferes with the infant’s usual activities). Furthermore, OH, TM, and GH scales were significantly but weakly and negatively correlated with IGSQ score. The weak correlation may be the result of the generally positive IGSQ scores in these infants, indicating minimal GI distress. These findings suggest that the parents of healthy children in China may not universally associate low intensity GI symptoms with their child’s health status (GD and BP) and may do so even less with their infant’s temperament and mood.

Despite the good health of the study population, it was possible to classify infants based on frequency of adverse events and number of HCP visits. Using these acute illness-related classifications, the ITQOL scales demonstrated sufficient discriminant validity. Infants who had one or more HCP visits had significantly worse scores for all ITQOL scales except PA, GD and FC. FC may not have been affected due to the availability of support from extended live-in family members. Additionally, the ITQOL was able to detect differences between subgroups of parents classified by their mental health status, providing further evidence of discriminative validity.

Although the PRO measures used in this study were shown to be age and culturally appropriate, mitigating the influence of the parent as the proxy-reporter on the evaluation of infant health-related outcomes such as HRQOL presents many challenges (Sherifali and Pinelli 2007; Civita et al. 2005). In the present study, the SF-12v2 was administered to account for the parents own mental well-being and to assess for confounding variables such as post-partum depression (Hays et al. 2016). In future studies, consideration should be given to the potential influence of socioeconomic status, spiritual beliefs, physical and environmental factors, and participant burden which may influence parents’ perceptions of infant HRQOL (Testa and Simonson 1996).

HRQOL PRO measures have the potential to provide valuable health information from the caregiver’s perspective. They may be used to estimate the burden of a disease, assess changes in health status, and compare the impact of different treatments on the child’s functional status and subjective well-being. The measurement of parent-reported infant HRQOL may facilitate parent engagement through parent-clinician dialogues and shared decision-making, and may one day be used for population surveillance, to advise public policy, and to prioritize and allocate healthcare resources.

There are several strengths to this study, namely, data were derived from a non-Western, community-based sample in a prospective study. Overall, the ITQOL performed well and there was good agreement between the ITQOL and other validated questionnaires. Limitations of this study include the observational design which utilized nonprobability sampling to enroll mostly healthy, first-born infants from single-child households living mainly in Eastern China. These characteristics of the study population, as well as the relatively small sample size in comparison to the population size of China, likely contribute to biases in the findings. A volunteerism effect also may have occurred, with parents of healthier infants more likely to participate and report positive health attributes. Thus it is unknown if these results would be replicated in a randomly-selected population including chronically ill infants. Also limiting the generalizability of the findings is the relative homogeneity of the sample with respect to marital status, educational level and presence of high levels of family support. Finally, study methodology limitations included that test/re-test reliability could not be calculated due to the length of time between study visits and PRO-related respondent burden on other study outcomes was not evaluated.

## Conclusion

Across 14 cities in Eastern, Central and Western China, the ITQOL translated into Simplified Chinese was shown to be feasible to administer in families with young infants. Study results suggests that the ITQOL is easy to complete, reliable and able to distinguish across acute illness-related classifications based on number of sick visits made to HCPs and occurrence of common childhood illnesses. Future work will focus on further aspects of instrument validity, reliability and sensitivity. Meanwhile, the analyses presented here suggest that the ITQOL may be a valuable tool for both researchers and clinicians to assess HRQOL in young Chinese infants.

## References

[CR1] Alonso EM, Neighbors K, Barton FB, McDiarmid SV, Dunn SP, Mazariegos GV (2008). Health-related quality of life and family function following pediatric liver transplantation. Liver Transplantation.

[CR2] American Academy of Pediatrics (1998). Guidelines for Health Supervision II.

[CR3] Bannink N, Maliepaard M, Raat H, Joosten KF, Mathijssen IM (2010). Health-related quality of life in children and adolescents with syndromic craniosynostosis. Journal of Plastic Reconstructive & Aesthetic Surgery.

[CR4] Caplan, F. (1988 and 1997). *The second twelve months of life: Your baby’s growth month by month*. New York: Bantam, Subsidiary of Putnam Publishing Group.

[CR5] Caplan, F., & Caplan, T. C. (1993 and 1995). *The first twelve months of life: Your baby’s growth month by month*. New York: Bantam Books.

[CR6] Cohen J (1988). Statistical power analysis for the behavioral sciences.

[CR7] Cronbach L (1951). Coefficient alpha and the internal structure of tests. Psychometrika.

[CR8] Darcy JM, Grzywacz JG, Stephens RL, Leng I, Clinch CR, Arcury TA (2011). Maternal depressive symptomatology: 16-month follow-up of infant and maternal health-related quality of life. Journal of the American Board of Family Medicine: JABFM.

[CR9] De Civita M, Regier D, Alamgir AH, Anis AH, Fitzgerald MJ, Marra CA (2005). Evaluating health-related quality-of-life studies in paediatric populations: Some conceptual, methodological and developmental considerations and recent applications. Pharmacoeconomics.

[CR13] Feng XL, Xu L, Guo Y, Ronsmans C (2012). Factors influencing rising caesarean section rates in China between 1988 and 2008. Bulletin of the World Health Organization.

[CR14] Festin MR, Laopaiboon M, Pattanittum P, Ewens MR, Henderson-Smart DJ, Crowther CA, SEA-ORCHID Study Group (2009). Caesarean section in four South East Asian countries: Reasons for, rates, associated care practices and health outcomes. BMC Pregnancy Childbirth.

[CR15] Flink IJ, Beirens TM, Looman C, Landgraf JM, Tiemeier H, Mol HA (2013). Health-related quality of life of infants from ethnic minority groups: The Generation R Study. Quality of Life Research.

[CR16] Hays NP (2016). Health-related quality of life among Chinese infants fed infant formula either exclusively or in combination with human milk. Health and Quality of Life Outcomes.

[CR17] HealthActCHQ. (2008). Confidential scoring rules, Infant Toddler Quality of Life Questionnaire. Boston, MA: HealthActCHQ.

[CR18] Landgraf JM, Abetz L (1994). The Infant/toddler quality of life questionnaire: Conceptual framework, logic, content, and preliminary psychometric results. Final Proprietiary Report to Schering-Plough Laboratories and Health Technology Associates.

[CR19] Lumbiganon P, Laopaiboon M, Gülmezoglu AM, Souza JP, Taneepanichskul S, Ruyan P, Attygalle DE, Shrestha N, Mori R, Nguyen DH, Hoang TB, Rathavy T, Chuyun K, Cheang K, Festin M, Udomprasertgul V, Germar MJ, Yanqiu G, Roy M, Carroli G, Ba-Thike K, Filatova E, Villar J, World Health Organization Global Survey on Maternal and Perinatal Health Research Group (2010). Method of delivery and pregnancy outcomes in Asia: The WHO global survey on maternal and perinatal health 2007-08. Lancet.

[CR22] Mao M, Zhang L, Ge J, Yan J, Northington R, Yao M (2018). Infant feeding regimens and gastrointestinal tolerance: A multicenter, prospective, observational cohort study in China. Global Pediatric Health.

[CR20] Mi J, Liu F (2014). Rate of caesarean section is alarming in China. Lancet.

[CR21] Norman GR, Sloan JA, Wyrwich KW (2003). Interpretation of changes in health-related quality of life: The remarkable universality of half a standard deviation. Medical Care.

[CR23] O’Hara MW, McCabe JE (2013). Postpartum depression: Current status and future directions. Annual Review of Clinical Psychology.

[CR24] Raat H, Landgraf JM, Oostenbrink R, Moll HA, Essink-Bot ML (2007). Reliability and validity of the Infant and Toddler Quality of Life Questionnaire (ITQOL) in a general population and respiratory disease sample. Quality of Life Research.

[CR25] Ravens-Sieberer U (2006). Generic health-related quality-of-life assessment in children and adolescents: Methodological considerations. Pharmacoeconomics.

[CR26] Riley AW, Trabulsi J, Yao M, Bevans KB, DeRusso PA (2015). Validation of a Parent Report Questionnaire: The Infant Gastrointestinal Symptom Questionnaire. Clinical Pediatrics.

[CR27] Saris-Baglama R, Dewey CJ, Chisholm GB, Plumb E, King J, Rasicot P (2010). Quality Metric Health Outcomes™, Scoring Software 4.0, User’s Guide.

[CR28] Sherifali D, Pinelli J (2007). Parent as proxy reporting: Implications and recommendations for quality of life research. Journal of Family Nursing.

[CR29] Spuijbroek AT, Oostenbrink R, Landgraf JM, Rietveld E, de Goede-Bolder A, van Beeck EF (2011). Health-related quality of life in preschool children in five health conditions. Quality of Life Research.

[CR30] Testa MA, Simonson DC (1996). Assesment of quality-of-life outcomes. New England Journal of Medicine.

[CR31] U.S. Department of Health and Human Services, Food and Drug Administration. (2015). Guidance for industry patient-reported outcome measures: Use in medical product development to support labeling claims. http://www.fda.gov/downloads/Drugs/GuidanceComplianceRegulatoryInformation/Guidances/UCM193282.pdf. Accessed July 15, 2015.

[CR32] Ware J, Kosinski M, Keller SD (1996). A 12-Item Short-Form Health Survey: Construction of scales and preliminary tests of reliability and validity. Medical Care.

[CR33] Wild D, Grove A, Martin M, Eremenco S, McElroy S, Verjee-Lorenz A (2005). Principles of good practice for the translation and cultural adaptation process for patient-reported outcomes (PRO) measures: Report of the ISPOR task force for translation and cultural adaptation. Value in Health.

[CR34] World Health Organization. (1948). Preamble to the Constitution of the World Health Organization as adopted by the International Health Conference, New York, 19–22 June, 1946; signed on 22 July 1946 by the representatives of 61 States (Official Records of the World Health Organization, 2, p. 100) and entered into force on 7 April 1948.

